# The Impact of Biological Sex, Medical School Background, and Geographic Location on Diversity in U.S. Neurosurgery Residency Programs

**DOI:** 10.7759/cureus.87051

**Published:** 2025-06-30

**Authors:** Maria Fioletova, Kassady A Perkinson, Minali Nemani, Julieanne P Sees

**Affiliations:** 1 Department of Medicine, Nova Southeastern University Dr. Kiran C. Patel College Of Osteopathic Medicine, Davie, USA; 2 Department of Medicine, Edward Via College of Osteopathic Medicine, Monroe, USA; 3 Health Sciences, Saint Joseph’s University, Erivan K. Haub School of Business, Philadelphia, USA

**Keywords:** allopathic, demographics, diversity, neurosurgery, osteopathic, residents, surgery

## Abstract

Background

Following an analysis of the National Residency Matching Program statistics, it was found that there is a ratio of 1:1.75 of candidates matching into a neurosurgical residency. The population of students accepted into neurosurgery residency programs appears less diverse compared to other surgical specialties. The objective of this descriptive, observational study is to examine the current trends among students accepted into neurosurgical residency programs and increase exposure to a more diverse demographic within the field of neurosurgery.

Methodology

A bibliometric analysis of neurosurgical residents across U.S. neurosurgical programs was conducted from 2017 to 2024. Data were collected from the Fellowship and Residency Electronic Interactive Database and residency websites between January and March 2024. Data were further divided into postgraduate years, biological sex, and medical degree.

Results

A total of 1,608 neurosurgical residents from 116 residency programs were identified. A decreasing trend in osteopathic residents was found from 4.29% of residents graduating in 2025 to 2.12% graduating in 2031. A steady trend was discovered in the percentage of women residents, increasing from 23.33% for residents graduating in 2025 to 30.51% for those graduating in 2031. The highest percentage of osteopathic residents in neurosurgery programs was found in the West Pacific area (8.3%, n = 17), the second highest in the Middle Atlantic (7.8%, n = 26), and the third highest in the West South Central (6.7%, n = 13). The highest percentage of female residents was found in the New England area (37.1%, n = 47), the second highest in the Pacific region (34.1%, n = 68), and the third highest in the South Atlantic (24.7%, n = 80).

Conclusions

The number of osteopathic residents in neurosurgery programs suggests a potential decline, which could be attributed to geographical, academic, and other factors. The number of female residents suggests a gradual increase; however, future steps toward addressing the gender disparities should be taken.

## Introduction

According to the 2024 National Resident Matching Program, for 241 positions offered, there were 423 applicants, resulting in a 1.75 ratio per spot [1]. The competitive nature of neurosurgery residency programs is well known; however, the lack of diversity within this field may not be widely recognized. Therefore, it is essential to comprehend the characteristics of the neurosurgery residency workforce. According to Accreditation Council for Graduate Medical Education (ACGME) data, in the 2023-2024 academic year, neurosurgery residency had the second lowest percentage of female residents (25.6%), with orthopedic surgery having the lowest percentage (22.4%) [2]. The low representation of female residents in these programs could be attributed to various factors such as low representation of women in faculty, barriers to career advancement, and lack of flexibility [3]. As for the low representation of osteopathic residents, there are several barriers, such as a lack of home programs, limited research opportunities, and the geographical distribution of programs.

The purpose of this study is to explore those who matched into neurosurgery residency programs in the United States from 2017 to 2024 and identify trends in the characteristics of the successfully matched students. Certain trends in the typical student profile have not been previously reported in those who successfully matched into one of these competitive residency programs. Factors such as biological sex, geographic location, and medical school reputation are important considerations with the ultimate aim of maintaining quality candidate interest while also making this field more diverse across our diverse nation.

## Materials and methods

Demographic data and bibliometric information were collected on current neurosurgical residents graduating between 2024 and 2031 across all U.S. ACGME-accredited residency programs. The study was conducted during the 2024-2025 academic year. Inclusion criteria encompassed all neurosurgical residents listed on official program websites and in the Fellowship and Residency Electronic Interactive Database (FREIDA). Programs without publicly available resident data were excluded from the study. Data collection involved manually extracting resident information from the official residency websites and FREIDA. These were accessed between January and March 2024. The residency program’s official website was prioritized as the main data source in case of discrepancies.

The collected variables were the location, U.S. geographic region, and hospital category (university-based or community-based) of the program. Neurosurgical residents were further categorized based on their degree, biological sex, and postgraduate year to assess trends over the past seven years. The data collection was then independently verified by all authors for accuracy. Geographical heat maps with percentages of residents with osteopathic degrees (DO) and female neurosurgical residents were created using Adobe Illustrator. Heat maps were used solely for visual illustrations of the residents’ distributions by regions and were not linked to regression models. The data were analyzed using descriptive statistics and linear regression analysis using the Excel Analysis ToolPak to identify trends in the typical student profile of those accepted into neurosurgery residency. A p-value <0.05 was considered statistically significant. This study did not involve personal contact with the residents; therefore, this study did not require Institutional Review Board approval.

## Results

**Figure 1:**
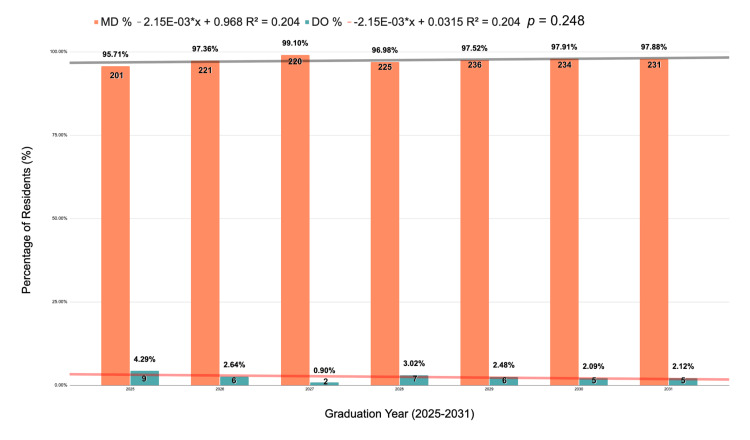
Percentages of osteopathic versus allopathic residents in Neurosurgery. Linear regression graph corresponding to osteopathic/DO vs. allopathic/MD neurosurgical residents from the 2025-2031 graduation year. Data shown as n (%). Statistical significance set at p < 0.05. The figure was created using Microsoft Excel.

A total of 1,608 neurosurgical residents from 116 residency programs were identified in the 2024-2025 academic year. A total of 97% (n = 1,568) of neurosurgical residents had an allopathic degree (MD), and 73% (n = 1,178) of residents were male. A decreasing trend was found in osteopathic residents from 4.29% (n = 9) of residents graduating in 2025, 2.64% (n = 6) in class of 2026, 0.9% (n = 2) in 2027, 3.02% (n = 7) in 2028, 2.48% (n = 6) in 2029, 2.09% (n = 5) in 2030, and 2.12% (n = 5) graduating in 2031 (R² = 0.204, p = 0.309). The lowest percentage of DO residents was found in the 2027 graduation year, with only two (0.9%) residents (Figure 1).

**Figure 2:**
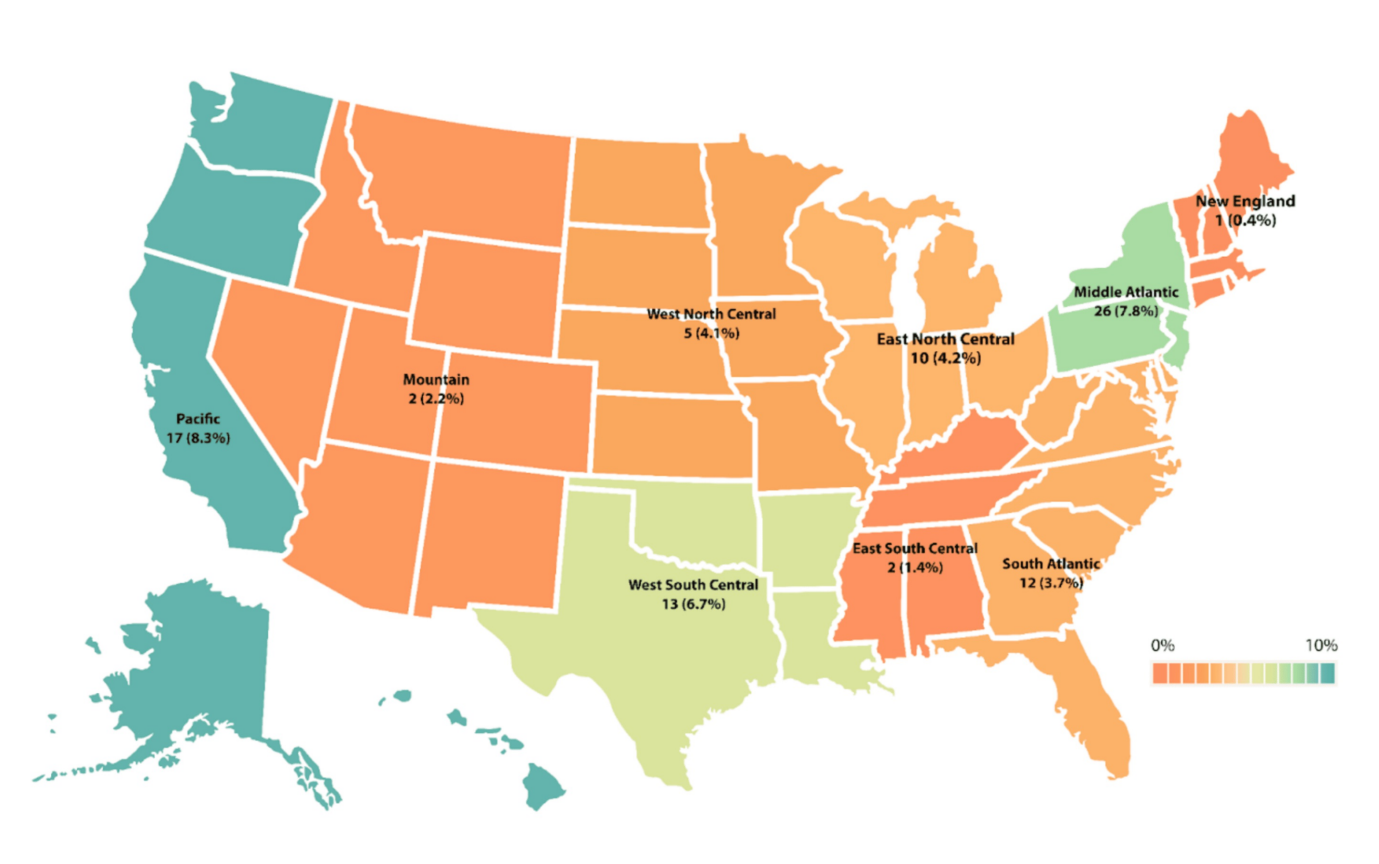
Percentages of osteopathic physicians in neurosurgical residency programs per region. The dark green color corresponds to the highest percentage of DO residents, and the dark red color corresponds to the lowest percentage. Values are labeled as n (%). Each region corresponds to the U.S. states mentioned below. New England: Connecticut, Maine, Massachusetts, New Hampshire, Rhode Island, Vermont. Middle Atlantic: New Jersey, New York, Pennsylvania. East North Central: Illinois, Indiana, Michigan, Ohio, Wisconsin. West North Central: Iowa, Kansas, Minnesota, Missouri, Nebraska, North Dakota, South Dakota. South Atlantic: Delaware, District of Columbia, Florida, Georgia, Maryland, North Carolina, Puerto Rico, South Carolina, Virginia, West Virginia. East South Central: Alabama, Kentucky, Mississippi, Tennessee. West South Central: Arkansas, Louisiana, Oklahoma, Texas. Mountain: Arizona, Colorado, Idaho, Montana, Nevada, New Mexico, Utah, Wyoming. Pacific: Alaska, California, Hawaii, Oregon, Washington. Data shown as n (%). The figure was created using Adobe Illustrator.

Further, neurosurgical residents were analyzed based on the geographical region in the United States. A total of nine geographical regions were included in the study, and all of them showed some percentage of osteopathic residents. The highest percentage of osteopathic residents in neurosurgery programs was found in the West Pacific area (8.3%, n = 17), the second highest in the Middle Atlantic (7.8%, n = 26), and the third highest in the West South Central (6.7%, n = 13). The rest of the regions were distributed as follows: East North Central (4.2%, n = 10), West North Central (4.1%, n = 5), South Atlantic (3.7%, n = 12), Mountain (2.2%, n = 2), East South Central (1.4%, n = 2), and the lowest percentage was found in the New England region (0.4%, n = 1) (Figure 2).

**Figure 3:**
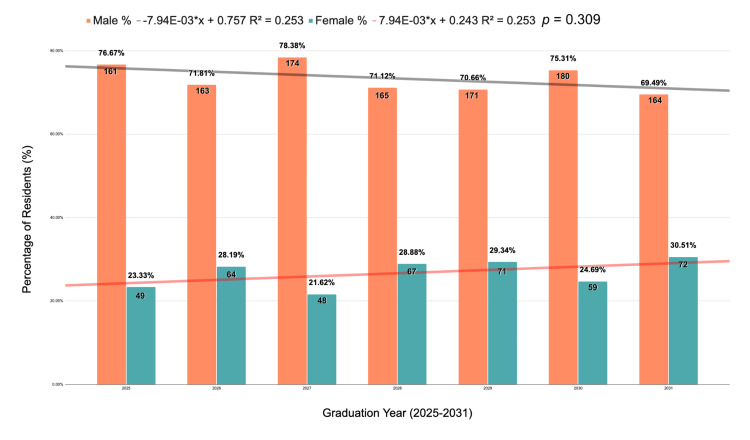
Percentages of female versus male residents in neurosurgery. Linear regression graph corresponding to female versus male neurosurgical residents from the 2025-2031 graduation year. Data shown as n (%). Statistical significance set at p < 0.05. The Figure was created using Microsoft Excel.

Female neurosurgical residents were analyzed in the past seven years (Figure 3). A steady trend was discovered in the percentage of women residents, increasing from 23.33% (n = 49) for residents graduating in 2025, 28.19% (n = 64) in 2026, 21.62% (n = 48) in 2027, 28.88% (n = 67) in 2028, 29.34% (n = 71) in 2029, 24.69% (n = 59) in 2030, and the highest percentage of 30.51% (n = 72) for those graduating in 2031 (R^2^ = 0.253, p = 0.248).

**Figure 4:**
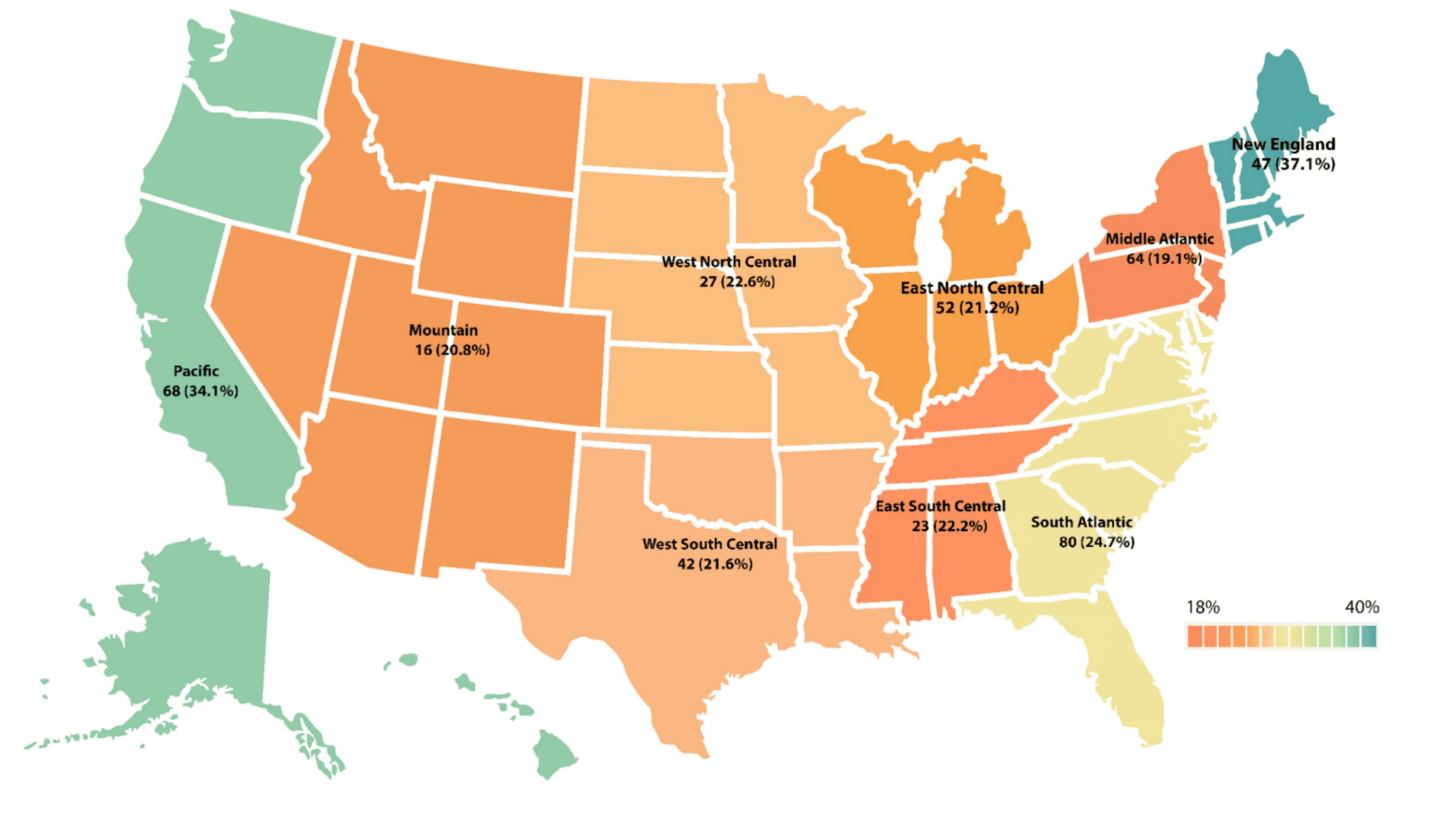
Percentages of female physicians in neurosurgery residency programs per region. The dark green color corresponds to the highest percentage of female residents (37%) in the New England area. The dark red color corresponds to the lowest percentage of 19.1% in the Middle Atlantic area. Values are labeled as n (%). Data shown as n (%). The figure was created using Adobe Illustrator.

Female neurosurgical residents were further analyzed based on geographical region in the United States (Figure 4). The highest percentage of female residents was found in the New England area (37.1%, n = 47), the second highest in the Pacific region (34.1%, n = 68), and the third highest in the South Atlantic (24.7%, n = 80). The rest of the regions were distributed as follows: West North Central (22.6%, n = 27), East South Central (22.2 %, n = 23), West South Central (21.6%, n = 42), East North Central (21.2%, n = 52), Mountain (20.8%, n = 16), and the lowest percentage was found in the Middle Atlantic (19.1%, n = 64).

## Discussion

This study considered the resident match data for neurosurgery residency classes 2024-2031. This study aims to identify trends in diversity within neurosurgery residency programs. The three main characteristics were biological sex, geographic location, and medical institution attended. These characteristics were chosen due to the vast majority of neurosurgery residents being male students from allopathic medical schools. Each of these characteristics is discussed below with possible explanations and solutions for the lack of diversity within these neurosurgery residency programs.

Our data show there has been a steady increase in the percentage of female neurosurgery residents. In the class of 2025, the percentage of female residents graduating from a neurosurgery residency program was 23.33% of all neurosurgery residency programs. In the class of 2031, the percentage of female residents graduating from a neurosurgery residency program was 30.51% of all neurosurgery residency program graduates. This is a drastic increase; however, the field of neurosurgery remains heavily male-dominated, with 69.49% of students accepted into the class of 2031 neurosurgery residencies being male.

Biological sex differences

It has been seen in other competitive surgical specialties that when there is a clear male dominance, there is an evident difference in the number of surgical cases between the sexes, with women getting fewer cases than men [4]. However, a study examining two programs in California, Riverside University Health System and Desert Regional Medical Center, did not reveal a discrepancy between females and males in the number of cases logged in those specific neurosurgery residencies. Biological sex disparity does not exist once females are accepted into these programs, yet there is still a disparity between the number of males versus females accepted into them [4].

One proposed explanation was potential disparities in research opportunities and pre-residency publications between females and males, leading to more match success in neurosurgical residency programs for men as opposed to women [5]. A cross-sectional analysis was performed and found that “there is no gender disparity in pre-residency publications among neurosurgery residents. To improve women’s representation in the field, further study is needed to better understand gender inequality among neurosurgeons, particularly in the earlier stages of medical training” [5]. Another proposed explanation for the difference in matched applicants was in the quality of their letters of recommendation. In the past, it was observed that there was a bias between letter writers and the students for whom they were writing letters. However, now it seems that the bias is no longer seen, and there are many more similarities between biological sexes in letters of recommendation for neurosurgical residencies [6].

The literature suggests that the lack of women in neurosurgical residency programs is due to the number of applications, not their quality. In a 2020 survey, it was found that “female neurosurgeons rated their career fulfillment worse than did male neurosurgeons (P < 0.001) and were less likely to choose a career as a neurosurgeon again (P < 0.001)” [7]. It was also found that “female neurosurgeons were less likely to be married or to have children than were male colleagues (P < 0.001)” [7]. The reality is that many women feel the need to choose between their careers and having a family when considering career paths such as neurosurgery. Ultimately, if the goal is to increase the number of female neurosurgeons and make this a more diverse workforce to treat diverse populations, then the focus, as many fields recognize, should be directed toward taking measures that improve the well-being of the healthcare provider, including work and family life balance, within this specialty and ultimately lead to higher job satisfaction [8].

This central theme of women being hesitant to pursue a neurosurgical career has led to there being a trend in certain geographic locations within the United States accepting more females into neurosurgery residency programs than in other parts of the United States. It was found that there were more female neurosurgeons located in urban states, and the greatest increase in female neurosurgeons was seen in rural states [9]. Urban states tend to have more training programs, more female physicians in general, and better policies relating to paternal leave [9]. Within rural states, there is a larger demand for neurosurgeons in general, which has led to this increase in female neurosurgeons in those states [9].

Osteopathic versus allopathic differences

Geographic trends in male versus female were not the only trends identified in neurosurgery residency program acceptances. There were also geographic trends in osteopathic (DO) versus allopathic (MD) students being accepted to these programs. A retrospective study analyzing data from candidates between 2018 and 2020 concluded that the MD match rates were more than three times higher than in DOs, with 36,194 students matching compared to only 10,659, respectively. Furthermore, these disparities were more present when looking at the competitiveness of the residency specialties, with match rate differences of only 4% in lowly competitive fields and a match rate difference of over 25% in highly competitive cohorts [10].

According to the analyzed data, for the class of 2025 through the class of 2031, a steady trend was found in the number of DOs accepted into neurosurgery residency programs, decreasing from 4.29% of residents graduating in 2025 to 2.12% graduating in 2031. The highest percentage of DO residents was found in the West Pacific area (8.3%). There are several possible explanations for why this trend is occurring, including a lack of research opportunities at osteopathic medical schools and a lack of a home program advantage.

A study in 2019 suggested a correlation between publication volume and matching into top neurosurgery residency programs given “Interns at top-25 neurosurgery residency programs tended to have a higher number of publications (8.3 ± 1.2 vs 4.8 ± 0.7, P = 0.0137), a higher number of neuroscience-related publications (6.8 ± 1.1 vs 4.1 ± 0.7, P = 0.0419), and a mean number of citations per publication (9.8 ± 1.7 vs 5.7 ± 0.8, P = 0.0267) compared to interns at all other programs” [11]. Research productivity accentuates the differences among osteopathic candidates compared to their allopathic peers, who may have more opportunities and funding. This can be seen in a study analyzing competitive research grants, concluding that the majority of these grants were allocated to allopathic physicians (78.03%), which contributed to more publications than osteopathic physicians [12].

Research publication opportunities and funding scarcities are not the only possible explanations for the lack of osteopathic physicians accepted into neurosurgery residency programs. Another possible explanation is the lack of neurosurgery residency programs at osteopathic medical schools. Most osteopathic medical schools do not have an academic hospital associated with their institution, which often leads to DO students rotating during their third and fourth years in community hospitals that lack neurosurgery residency programs or even a neurosurgery service. This results in a large number of DO students who may be interested in neurosurgery being deficient in early exposure to the field, with their only encounter during the fourth year, when the focus is on audition rotations in previously encountered fields of surgery and medicine.

These exposure deficits before or even during clinical rotations often lead to neurosurgery not even being considered by osteopathic students. It was found that most students think positively of the field of neurosurgery, and interest could be strengthened by increasing exposure to the field by either making it a core rotation for third years or ensuring rotation sites include this surgical specialty [13]. Another factor recognizing home programs lacking this surgical specialty at osteopathic medical schools may explain both the lower numbers of osteopathic students at neurosurgical residency as well as the geographical trends of those accepted.

Geographic location

Our study found a correlation between the location of the program and the medical schools that the residents attended. In 2015, a study showed that this preference toward applicants who attended medical schools within the same region was present across all surgery residency programs [14]. In the 2021 graduate cycle, the top 40 National Institutes of Health (NIH)-funded schools’ graduates were matched closer to their home institution. This could be due to NIH-funded medical schools having robust residency options that appeal to internal applicants [15].

The geographic distribution of DO neurosurgery residents is highly concentrated in New Jersey (n = 11, 27.5%), Michigan (n = 9, 17.3%), Arkansas (n = 1, 14.29%), and South Carolina (n = 2, 14.29%) [16]. These trends could be attributed to regional and institutional preferences of osteopathic students for formerly American Osteopathic Association-accredited programs [16]. Another reason for the high concentration of DO residents in those areas could be that residents stay in the same geographical area upon completion of training due to formed connections and living arrangements. Overall, the limited number of neurosurgical programs that would attract candidates leads to areas having an inherently low number of neurosurgeons [17].

Study limitations

There are several limitations to our study. Our data were limited to the publicly available data from the residency websites and the FREIDA website, which may not fully capture neurosurgical residents’ demographics. The data extraction was conducted by the authors following standardized procedures, but inter-rater reliability was not assessed, which may lead to selection bias. The data provided on the websites may have limitations, such as an inability to capture ethnicity, race, previous educational experience, age, and many other characteristics that could affect the dataset. Due to the limited publicly available data, we have not conducted sensitivity analyses. Despite the limitations, the data are consistent with the publicly available information. Lastly, given the low R² values and high p-values, our trend analysis should be interpreted as a way to identify general trends rather than to predict outcomes.

## Conclusions

Our analysis of neurosurgery residency data suggests a consistent trend in acceptance rates with diversity related to biological sex, geographic location, and medical institution background. Despite seeing a gradual increase in female representation in neurosurgery programs, the predominance of men and allopathic medical doctors is still significant. These could be attributed to factors such as work-life balance concerns, geographic trends, and disparities in opportunities within different institutions. Our findings suggest that the diversity in neurosurgery residency programs should be addressed to better match the diversity of the nation’s population. This can be accomplished by increasing mentorship efforts and research opportunities and reducing the disparities between allopathic and osteopathic students. Further research can expand on the disparities regarding biological sex and medical institutions, focusing on the availability of research and mentorship programs. Investigating these can contribute to finding concrete solutions to these disparities.
